# Supplementary material for: High-Dose Spermidine Supplementation Does Not Increase Spermidine Levels in Blood Plasma and Saliva of Healthy Adults: A Randomized Placebo-Controlled Pharmacokinetic and Metabolomic Study
Source: Nutrients. 2023 Apr 12;15(8):1852. doi: 10.3390/nu15081852 (PMC10143675; doi:10.3390/nu15081852)
Supplement: Supplementary file 1 [file nutrients-15-01852-s001.zip › nutrients-2279215-supplementary.pdf]

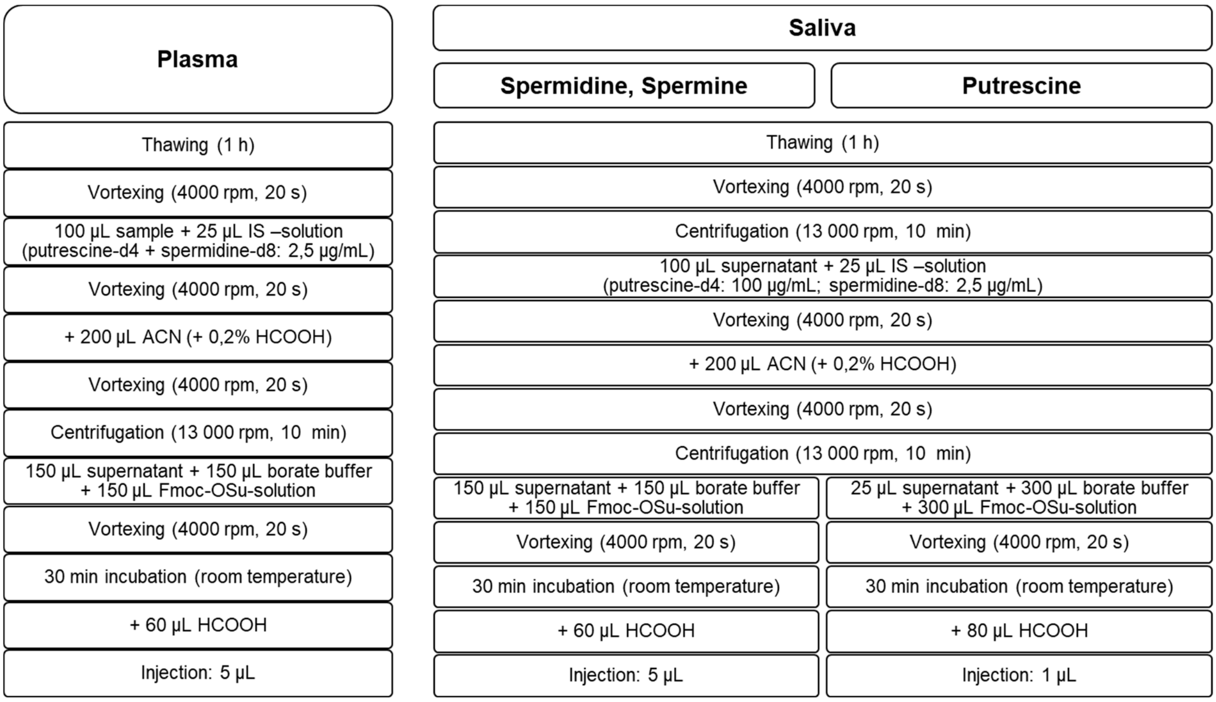

Figure S1: Overview of the steps for the preparation of plasma and saliva samples for LCMS analysis. (IS – internal standard, ACN – acetonitrile, Fmoc-OSu – N-(9-Fluorenylmethoxycarbonyloxy)succinimide).

Table S1: Gradient conditions for the used LCMS method for the determination of polyamines.

Time (min)	Eluent A (%)	Eluent B (%)
0.0	40.0	60.0
5.5	5.0	95.0
10.0	5.0	95.0
10.1	40.0	60.0

Table S2: MRM transitions used for quantification with the corresponding collision energies.

Compound	MRM-transition	Collision energy (eV)
Putrescin	533.40 → 337.10	-12.0
Spermidine	590.40 → 179.00	-42.0
Spermine	869.50 → 179.05	-54.0
d4-Putrescine	537.50 → 341.10	-11.0
d8-Spermidine	598.60 → 179.10	-43.0

Table S3: Immediate and Intermediate Accuracy and Precision of the determination of polyamines in human plasma.

Compound	Qualitiy control level	Nominal concentration (ng/mL)	Intra-day (n=6)		Inter-day (n=18)	
			Accuracy (%)	RSD (%)	Accuracy (%)	RSD (%)
Putrescine	QC-L	5	102.35	5.01	96.81	17.54
	QC-M	150	103.43	3.94	99.19	6.27
	QC-H	375	103.05	2.65	99.14	5.64
Spermidine	QC-L	5	106.91	4.18	102.42	12.66
	QC-M	150	103.22	3.79	100.18	6.90
	QC-H	375	99.32	1.51	97.66	5.80
Spermine	QC-L	5	98.31	14.11	95.88	17.06
	QC-M	150	107.76	5.44	103.16	9.45
	QC-H	375	111.75	4.43	111.04	9.38

Table S4: Immediate and Intermediate Accuracy and Precision of the determination of polyamines in human saliva.

Compound	Qualitiy control level	Nominal concentration (µg/mL)	Intra-day (n=6)		Inter-day (n=18)	
			Accuracy (%)	RSD (%)	Accuracy (%)	RSD (%)
Putrescine	QC-L	1	113.66	6.78	103.05	10.27
	QC-M	30	112.68	4.38	105.21	6.04
	QC-H	75	112.39	4.48	109.19	3.53
Spermidine	QC-L	25	111.53	7.61	108.52	8.46
	QC-M	750	101.75	1.20	103.28	2.59
	QC-H	1875	98.34	4.64	101.57	4.53
Spermine	QC-L	25	98.57	7.68	87.57	16.12
	QC-M	750	104.04	2.15	99.33	10.21
	QC-H	1875	114.59	2.38	108.47	11.76

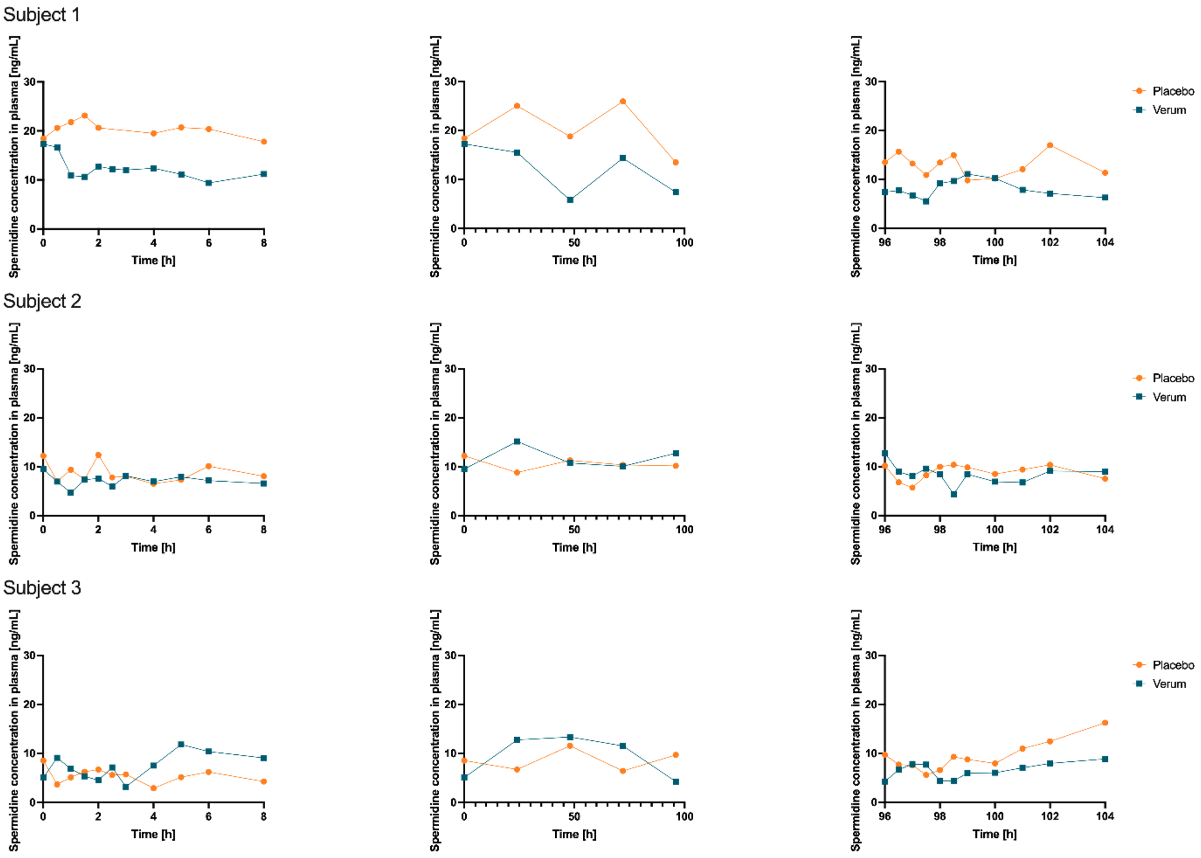

Figure S2: Individual graphs of subjects 1, 2 and 3 for plasma spermidine in both the verum and placebo intervention; divided into respectively left: 0-8 h after administration, middle: all fasted matutinal samples, right: 96-104 h of the intervention phases.

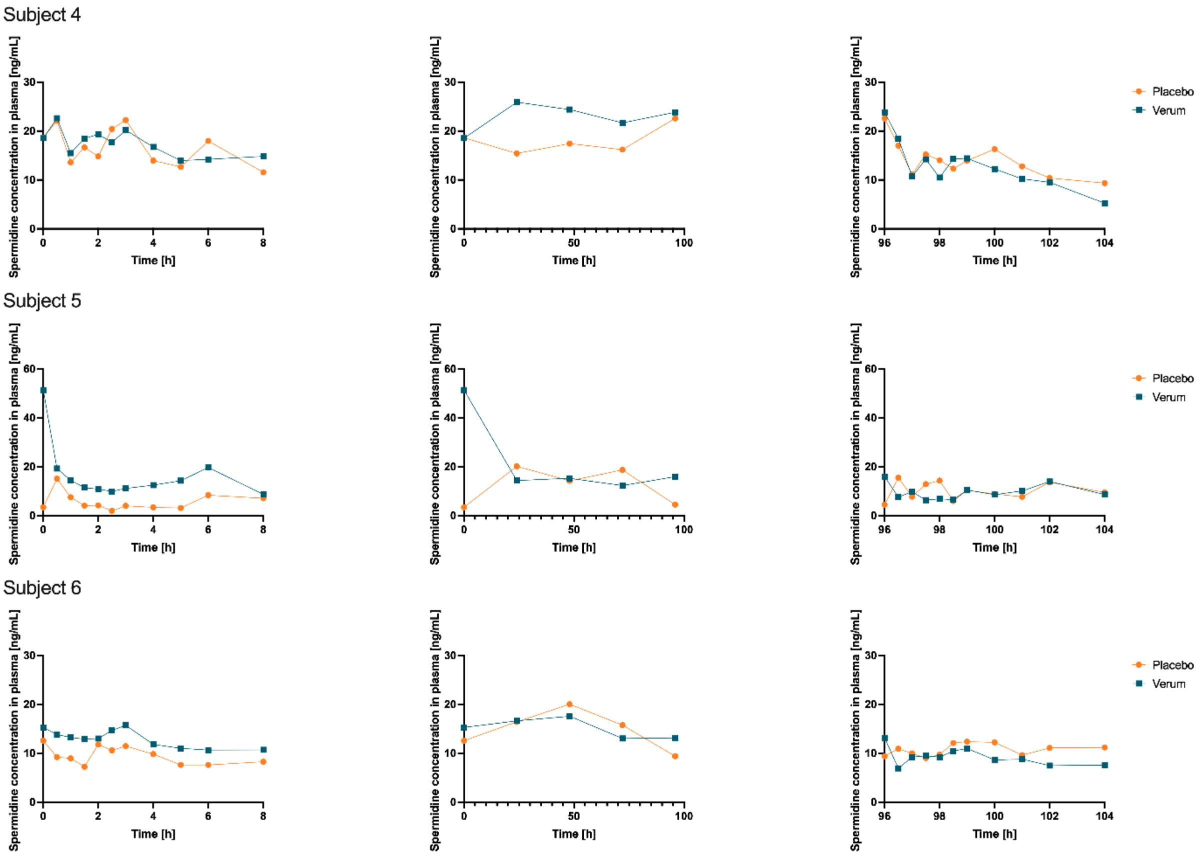

Figure S3: Individual graphs of subjects 4, 5 and 6 for plasma spermidine in both the verum and placebo intervention; divided into respectively left: 0-8 h after administration, middle: all fasted matutinal samples, right: 96-104 h of the intervention phases. Note: different y-axis subject 5.

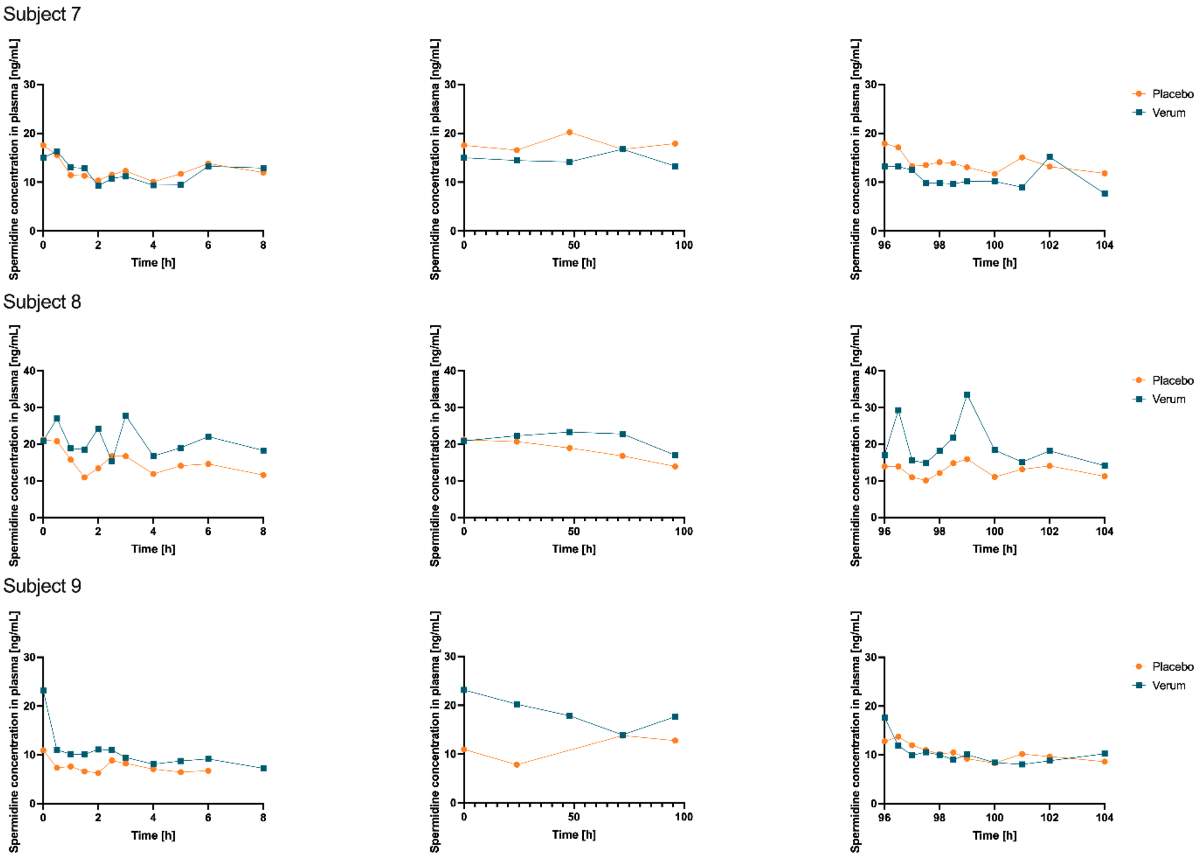

Figure S4: Individual graphs of subjects 7, 8 and 9 for plasma spermidine in both the verum and placebo intervention; divided into respectively, left: 0-8 h after administration, middle: all fasted matutinal samples, right: 96-104 h of the intervention phases. Note: different y-axis subject 8.

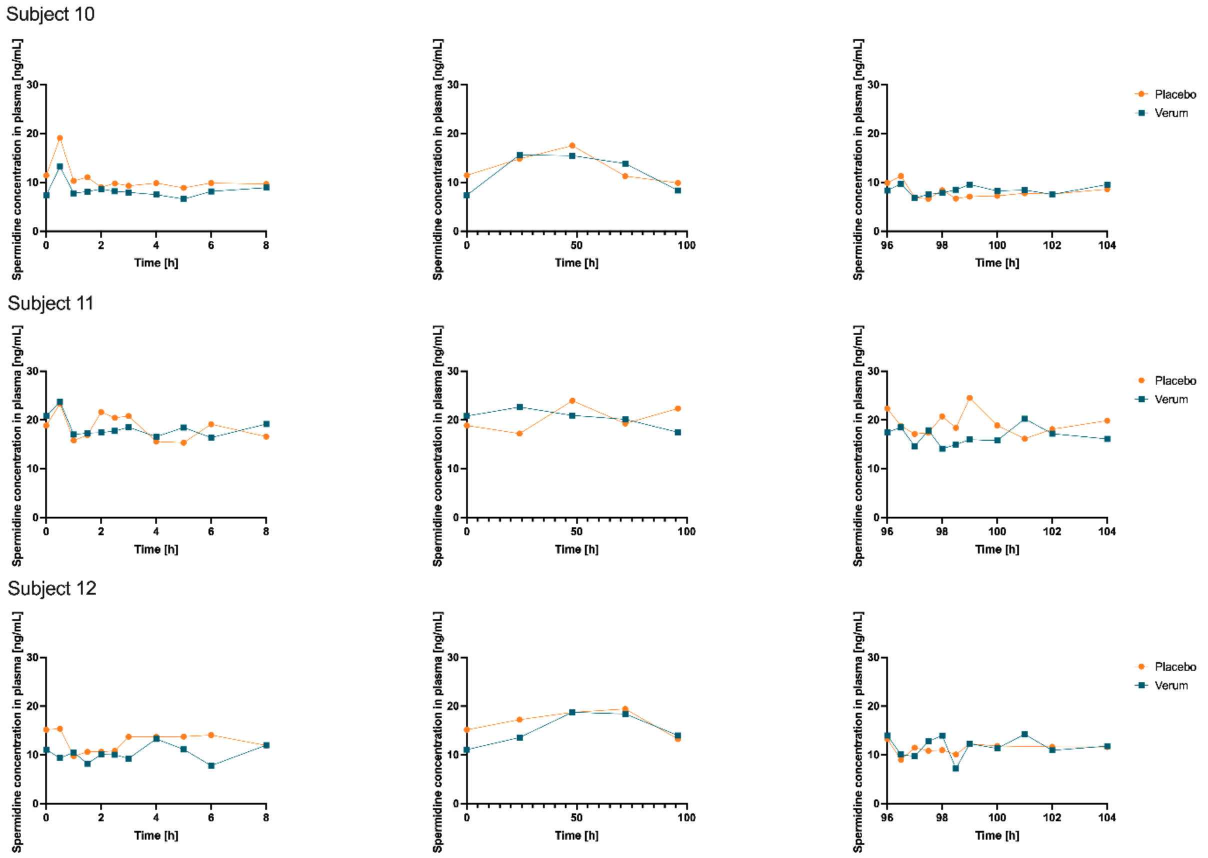

Figure S5: Individual graphs of subjects 10, 11 and 12 for plasma spermidine in both the verum and placebo intervention; divided into respectively, left: 0-8 h after administration, middle: all fasted matutinal samples, right: 96-104 h of the intervention phases.

Table S5: Individual AUC 0-tlast, cmax, tmax and mean p-values for spermidine plasma in both verum and placebo intervention

Parameter	Subject	Placebo	Verum	Statistics (p-value)
AUC 0-tlast (h• ng/mL)	1	2147	1132	0.7819 (ns)
	2	1022	1131	
	3	841.2	1098	
	4	1715	2255	
	5	1435	1407	
	6	1528	1481	
	7	1736	1476	
	8	1752	2225	
	9	1094	1594	
	10	1331	1323	
	11	2082	2081	
	12	1711	1604	
cmax (ng/mL)	1	25.98	17.28	0.7754 (ns)
	2	12.4	15.16	
	3	16.26	13.39	
	4	22.59	25.89	
	5	20.2	51.37	
	6	20.04	17.6	
	7	20.23	16.75	
	8	21.25	33.6	
	9	13.78	23.15	
	10	19.13	15.65	
	11	24.53	23.74	
	12	19.43	18.77	
tmax (h)	1	72	0	0.2317 (ns)
	2	2	24	
	3	104	48	
	4	96	24	
	5	24	0	
	6	48	48	
	7	48	72	
	8	0	99	
	9	72	0	
	10	0,5	24	
	11	99	0.5	
	12	72	48	

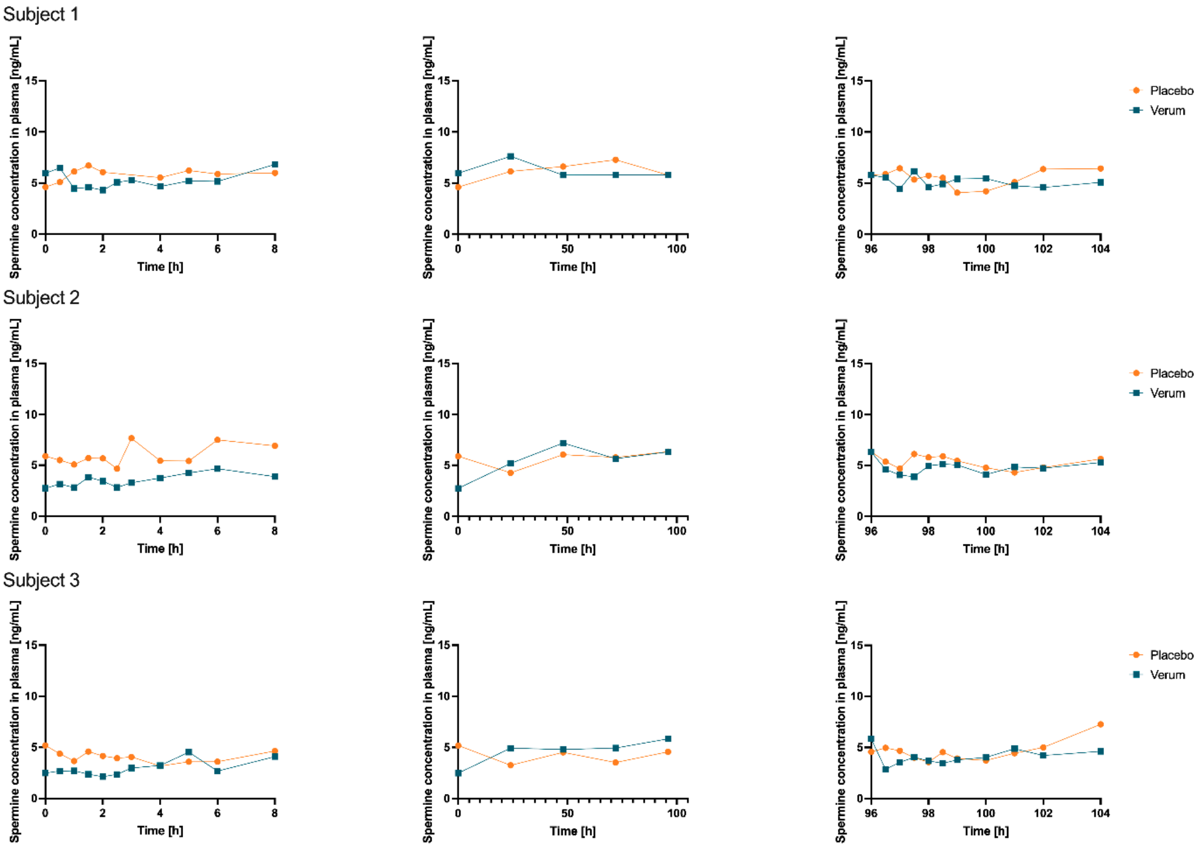

Figure S6: Individual graphs of subjects 1, 2 and 3 for plasma spermine in both the verum and placebo intervention; divided into respectively, left: 0-8 h after administration, middle: all fasted matutinal samples, right: 96-104 h of the intervention phases.

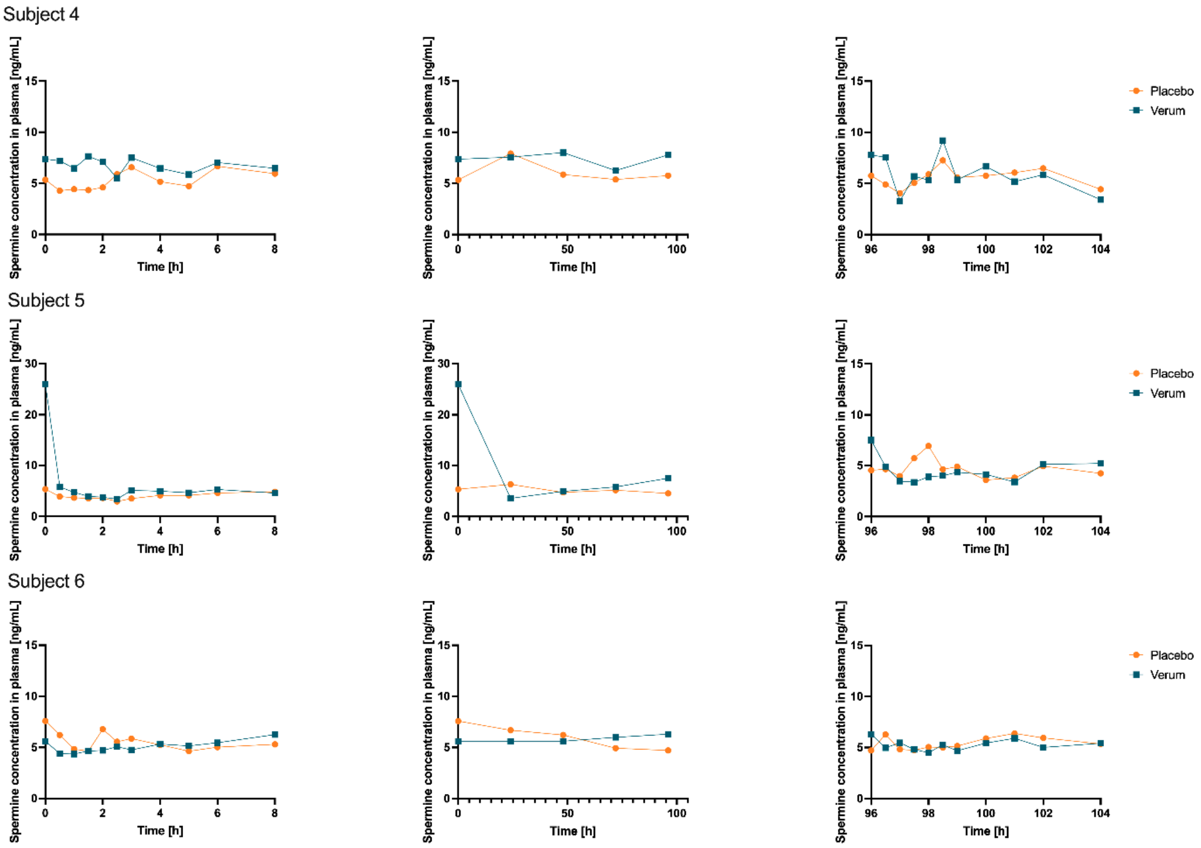

Figure S7: Individual graphs of subjects 4, 5 and 6 for plasma spermine in both the verum and placebo intervention; divided into respectively, left: 0-8 h after administration, middle: all fasted matutinal samples, right: 96-104 h of the intervention phases. Note: different y-axis subject 5.

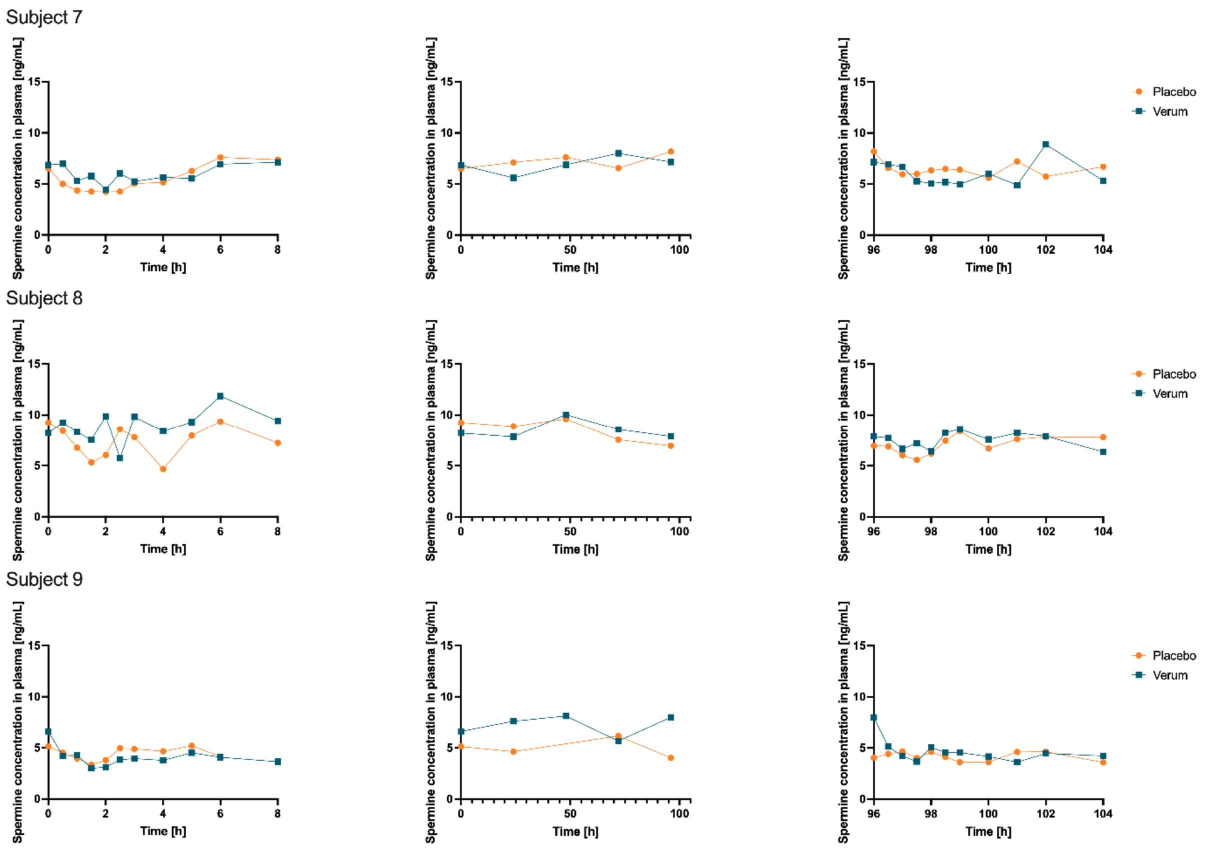

Figure S8: Individual graphs of subjects 7, 8 and 9 for plasma spermine in both the verum and placebo intervention; divided into respectively, left: 0-8 h after administration, middle: all fasted matutinal samples, right: 96-104 h of the intervention phases.

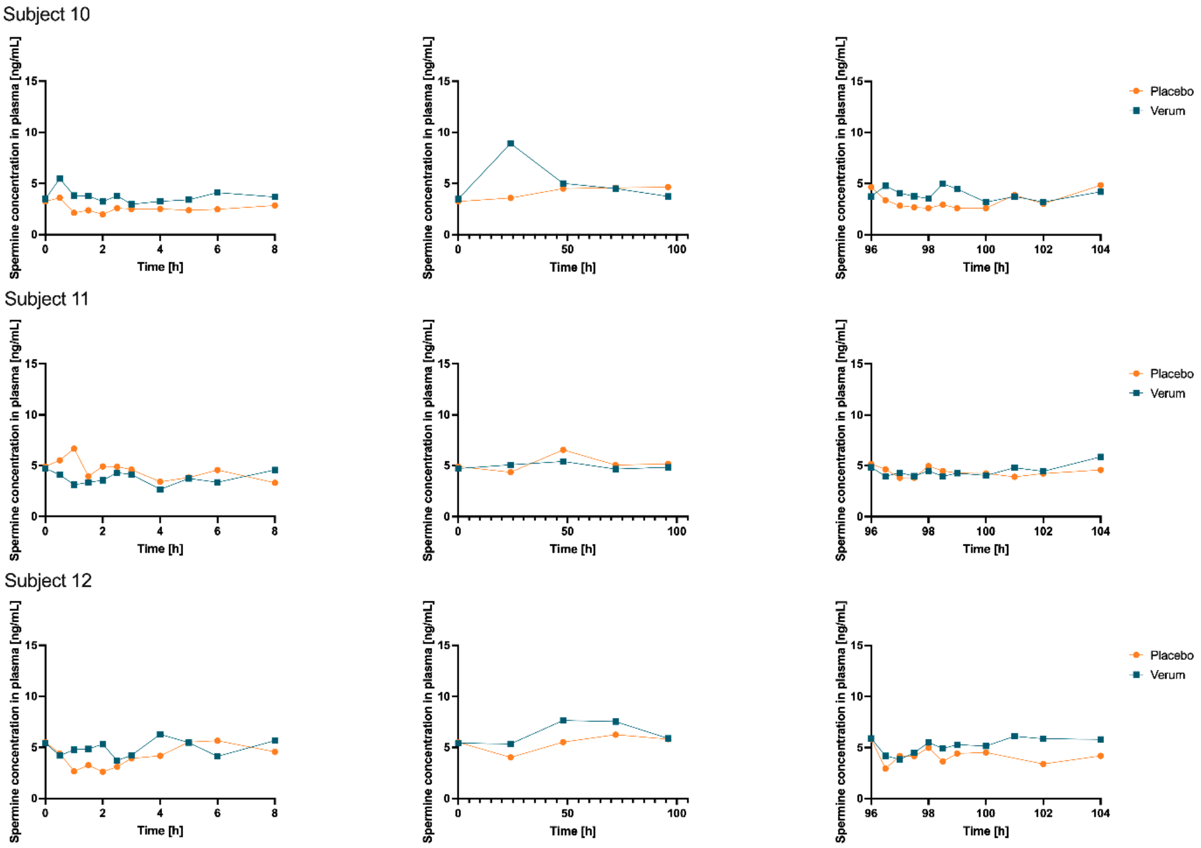

Figure S9: Individual graphs of subjects 7, 8 and 9 for plasma spermine in both the verum and placebo intervention; divided into respectively, left: 0-8 h after administration, middle: all fasted matutinal samples, right: 96-104 h of the intervention phases.

Table S6: Individual AUC 0-tlast, cmax, tmax and mean p-values for spermine plasma in both verum and placebo intervention

Parameter	Subject	Placebo	Verum	Statistics (p-value)
AUC 0-tlast (h• ng/mL)	1	665.2	637	0.0282 (s)
	2	590.6	585.8	
	3	419.6	493.1	
	4	634.1	738.3	
	5	524.8	533.4	
	6	588.1	599.8	
	7	737.3	710.4	
	8	848.4	908.4	
	9	521.8	676.1	
	10	415.1	541.8	
	11	522.5	502.4	
	12	537.7	669.9	
cmax (ng/mL)	1	7.279	7.615	0.1294 (ns)
	2	7.638	7.171	
	3	7.266	5.84	
	4	7.917	9.201	
	5	6.913	25.98	
	6	7.588	6.311	
	7	8.184	8.878	
	8	9.61	11.86	
	9	6.177	8.127	
	10	4.839	8.921	
	11	6.643	5.85	
	12	6.256	7.651	
tmax (h)	1	72	24	0.9983 (ns)
	2	3	48	
	3	104	96	
	4	24	98,5	
	5	98	0	
	6	0	96	
	7	96	102	
	8	48	6	
	9	72	48	
	10	104	24	
	11	1	104	
	12	72	48	

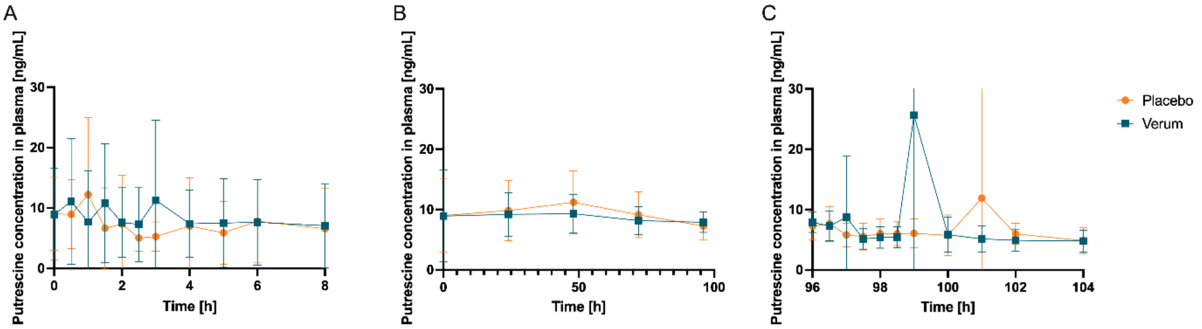

Figure S10: Concentrations of putrescine in plasma, mean and standard deviation all 12 subjects combined
A) Day 1 and day 15 (first measurement day of each intervention phase), 0-8 h after first administration of verum or placebo; B) first sample on days 1, 5, 15, 19 and days 2, 3, 4, 16, 17, 18 (all fasted matutinal samples of each intervention phase); C) day 5 and day 19 (last measurement day of each intervention phase), 96-104 h after multiple administration of verum or placebo.

Table S7: Plasma putrescine mean and SD AUC 0-tlast, cmax, tmax and p-value placebo versus verum.

Parameter	Placebo	Verum	Statistics (p-value)
AUC 0-tlast (mean + SD) h・ ng/mL	931.7 (392.7)	882.8 (266.2)	0.9097 (ns)
cmax (mean + SD) ng/mL	20.73 (18.90)	36.36 (64.63)	0.3394 (ns)
tmax (mean + SD) h	43.08 (43.27)	37.13 (40.08)	0.6543 (ns)

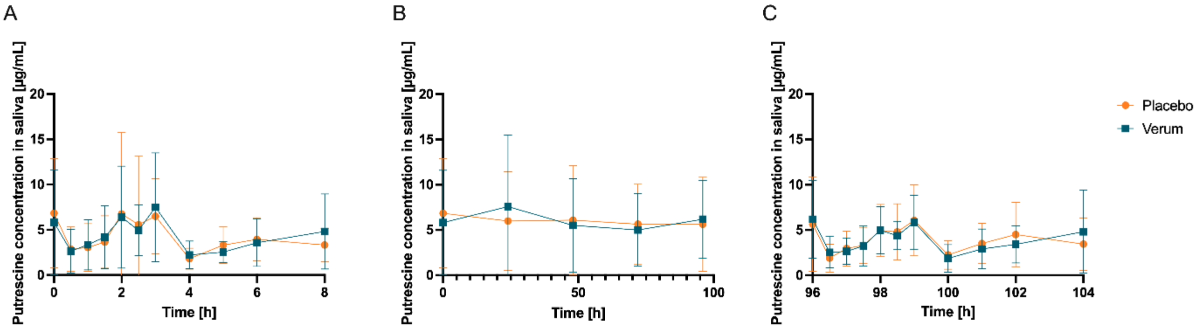

Figure S11: Concentrations of putrescine in saliva, mean and standard deviation of all 12 subjects combined
A) day 1 and day 15 (first measurement day of each intervention phase), 0-8 h after first administration of verum or placebo; B) first sample days 1, 5, 15, 19 and days 2, 3, 4, 16, 17, 18 (all fasted matutinal samples of each intervention phase); C) days 5 and day 19 (last measurement day of each intervention phase), 96-104 h after multiple administration for verum or placebo.

Table S8: Saliva putrescine mean and SD AUC 0-tlast, cmax, tmax and p-value in both verum and placebo intervention.

Parameter	Placebo	Verum	Statistics (p-value)
AUC 0-tlast (mean + SD) h・ µg/mL	568.8 (424.0)	578.3 (418.3)	0.3394 (ns)
cmax (mean + SD) µg/mL	11.69 (8.849)	12.03 (8.281)	0.7002 (ns)
tmax (mean + SD) h	47.58 (44.87)	49.71 (43.93)	0.9658 (ns)

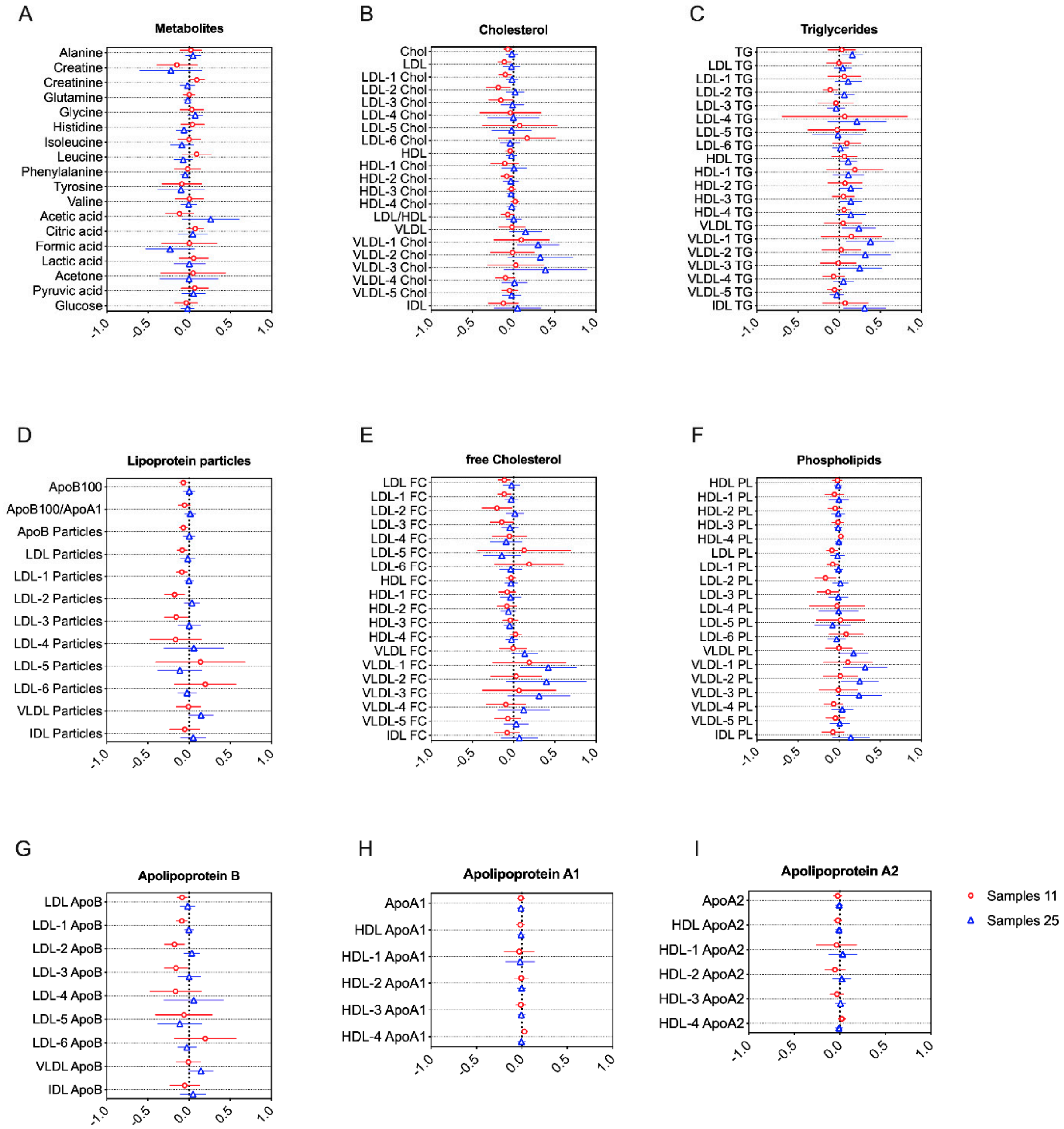

Figure S12: Forest plot based on the relative error with placebo sample as reference value.
A) Metabolites; B) Cholesterol; C) Triglyceride; D) Lipoprotein particles; E) Free Cholesterol; F) Phospholipide; G) Apolipoprotein B; H) Apolipoprotein A1; I) Apolipoprotein A2.
